# Development of a theoretically based implementation protocol

**DOI:** 10.1186/1472-6963-14-S2-P83

**Published:** 2014-07-07

**Authors:** Joanna Moullin, Daniel Sabater-Hernández, Shalom Benrimoj

**Affiliations:** 1 Graduate School of Health, University of Technology Sydney, Sydney, NSW, Australia

## Background

Internationally there is a gap between the development and delivery of professional pharmacy services. The fields of implementation science and knowledge translation have developed across disciplines with the objective of bringing evidence to practice. What appears necessary for success, but is not evident in implementation frameworks, is that core concepts should be considered for every implementation effort. A Generic Implementation Framework (GIF) was developed to illustrate these core concepts [1]. The framework consists of six components; process of implementation, domains (the innovation to be implemented and the context in which the implementation is to occur), influencing factors, strategies and interventions utilised to aid the process, and evaluations employed. The GIF may be utilised as a base for the development of implementation protocols or programs and then tailored for use, depending on the innovation, user, setting, discipline and objective. The GIF was operationalized for community pharmacy as the Framework for the Implementation of Pharmacy Services (FISpH) and practically applied to design an implementation study.

## Methods

The FISpH framework was used to develop an implementation study protocol for a professional pharmacy service in Spain, medication review with follow-up. The implementation study is a hybrid design [2] consisting of a 3 month pilot and a 15 month main study in 100 pharmacies across 10 Spanish provinces.

## Results

Implementation strategies employed include interactive training sessions with pharmacy owners and service providers, monthly outreach facilitator visits and the assignment of an internal pharmacy champion to take charge of the implementation team as implementation progresses. The facilitators’ and champions’ role includes analyzing barriers and facilitators and subsequent tailoring of interventions to overcome or utilize the realized influencing factors. Outcomes to be measured are the movement of pharmacies through implementation stages, service benefits, reach, fidelity and integration.

## Conclusion

The Generic Implementation Framework appears to be an applicable base to tailor to pharmacy practice and subsequently develop implementation protocols. The consequent Framework for Implementation of Services in Pharmacy is well understood by stakeholders at policy, professional organization, pharmacy owner and employee staff levels.
